# Exploring the social and organizational factors influencing dog bites: a qualitative study

**DOI:** 10.1186/s12889-025-26083-9

**Published:** 2026-01-02

**Authors:** Vahid Nohtani, Mehrsadat Mahdizadeh, Mahdi Gholian-Aval, Vahid Ghavami, Hadi Tehran

**Affiliations:** 1 Department of Health Education and Health Promotion, School of Health, student research committee, Mashhad University of Medical Sciences, Mashhad, Iran; 2 Social Determinants of Health Research Center, Mashhad University of Medical Sciences, Mashhad, Iran; 3 Department of Health Education and Health Promotion, School of Health, Mashhad University of Medical Sciences, Mashhad, Iran; 4 Department of Biostatistics, School of Health, Mashhad University of Medical Sciences, Mashhad, Iran

**Keywords:** Dog management, Social factor, Qualitative study, Organizational factors

## Abstract

**Introduction:**

Dog bites, a public health problem of global concern, have been causing increasing worries in the health system. This study was conducted with the aim of identifying the social and organizational-political factors that influence dog bites in Khash County, Sistan and Baluchestan Province, Iran.

**Methods:**

This qualitative study delved into the factors affecting dog bites using guided content analysis. A purposive sampling of 41 participants (residents, bitten individuals, families, and institutional officials) was conducted between March 2025and June 2025. Data were collected through semi-structured interviews and analyzed with MAXQDA 12 software, ensuring a thorough understanding of the issue.

**Results:**

The factors were classified into four main categories at two social and organizational-political levels: Cultural factors include beliefs and attitudes in keeping semi-free dogs and irresponsibility of dog owners; Behavioral factors include risky behaviors, inappropriate interactions with dogs, and vulnerability of specific groups such as children and the elderly; organizational-political factors include inter-institutional incoherence and lack of sustainable funding; and facilitators include institutional capacities and social demands.

**Discussion:**

The findings underscore the need for integrated interventions to manage and prevent dog bites. These interventions, which should be implemented at both local and national levels, include local education in the Balochi dialect, strengthening inter-institutional coordination, and sustainable budget allocation. National policies can create a preventive environment to reduce the burden of this problem by empowering local communities, highlighting the importance of collaboration in addressing this issue.

**Supplementary Information:**

The online version contains supplementary material available at 10.1186/s12889-025-26083-9.

## Introduction

Dog bites have long been recognized as a public health problem for the serious infections and injuries they cause [1]. In the UK, the estimated rate of dog bites is much higher; around 1870 cases per 100,000 people, with around a quarter of people reporting having been bitten by a dog at least once in their lifetime [2]. In New Zealand, around 10,951 people (242 per 100,000 people) visit health facilities each year for dog bites, with people of lower socioeconomic status being more affected [3]. Recent statistics show an increase in dog bites in Iran between 1993 and 2020 [4–6]. In Iran, about $12 million is spent annually on rabies vaccines [7].

Dog bites are an important indicator of community safety. It is a key indicator because it describes both the effects on public health risks and the risks to livestock and wildlife, including biting and threatening behavior towards people and other animals. Dog bite injuries are one of the most important causes of morbidity, especially in children, leading to high rates of hospitalization and significant psychological distress even without the threat of rabies [3, 8]. Stray dogs impact society by restricting people’s freedom to move around their neighborhoods and preventing physical activity [9]. They also deface the city by scattering garbage in cities and villages, which in turn facilitates disease transmission. Despite the long history and efforts made by the healthcare system in animal bites, especially rabies, in Iran, there are challenges in managing the disease, and animal bite cases remain high, and Iran is an endemic region. Iran faces several challenges in controlling animal bites, including a lack of public awareness in rural areas, a lack of vaccination of pets, and the infiltration of stray dogs from neighboring countries, which exacerbates the risk of disease transmission, such as rabies, in border provinces [10]. These barriers, in complex interaction with social and organizational factors, deepen the challenges of controlling and preventing animal bites in Iran. Social factors, such as cultural norms and public awareness, drive risky human-dog interactions, while organizational factors, like inter-institutional coordination and resource allocation, shape prevention effectiveness [11, 12].

This study was designed to answer the following questions: (A) What are the social and cultural factors that explain dog bites? (B) What are the organizational and political factors that affect dog bites? (C) What are the policies in place to implement a dog bite prevention program?

Khash County, Sistan and Baluchestan, Iran, faces a high rate of dog bite incidents due to its unique border challenges. The number of cases rose from 287 in 2021 to 293 in 2022 and 312 in 2023 (an increase of 8.7%). The rates per 100,000 population were 148.5 in 2021 and 2023, and 132.9 in 2022 [11]. These rates exceed the national average for Iran (140–180 per 100,000) [8], and proximity to the Pakistani border increases risks through the infiltration of stray dogs [10].

### Social factors

Social factors refer to the set of conditions, norms, values, relationships, interactions, and structures existing in a society that directly or indirectly influence public health, health behaviors, and health-related outcomes. These factors include various aspects of social life, such as social networks, social support, cultural beliefs, socioeconomic inequalities, and collective norms that can shape or constrain the behaviors of individuals and groups in health contexts. Social factors are of particular importance in the field of public health due to their profound influence on risky or protective behaviors and their role in creating health inequalities [12].

### Organizational factors

Organizational factors refer to the policies, structures, processes, and resources available in organizations that influence service delivery, implementation of health interventions, and management of public health issues. These factors include inter-organizational coordination, access to resources, policy frameworks, and management capacities that shape the conditions and effectiveness of health programs. Organizational factors are of particular importance because of their role in determining the quality and access to health services, including the prevention and control of zoonotic diseases [13, 14].

In Iran and around the world, several qualitative studies have been conducted on dog bites and related factors. A study by Hatam et al. [15] in Shiraz identified lack of public awareness and poor education as key factors in rabies prevention. Westgarth et al. [16] also emphasize the role of public education and dog management in the UK. Saleem et al. [17] also examine educational barriers and the diversity of victims’ perceptions of street dogs in India, emphasizing public education. However, not only have these studies not addressed specific cultural norms, but none of them have utilized a comprehensive conceptual framework to analyze the interaction and combination of social and organizational-political factors. Therefore, this study contributes to our understanding in several ways. First, it provides a structured conceptual framework for the factors influencing dog bites. Second, it identifies the triggers for dog bites in the context of litter, which may apply to other regions of the world with similar sociocultural norms and boundary challenges.

## Methods

### Study design

In this study, we used Directed Content Analysis (DCA) because of its ability to use existing frameworks to guide the analysis of qualitative data and identify social and organizational factors influencing dog bites. This approach allows for systematic organization of data and is consistent with the cultural context of Khash and the need to identify institutional barriers and opportunities. The interpretivist/constructivist paradigm allowed us to explore social meanings and institutional structures in depth in the local context. This paradigm was chosen instead of a positivist approach because the study aims to explain subjective meanings and experiences rather than to measure variables [17] objectively. We followed the guidelines of the Standards for Qualitative Research Reporting (SRQR) [18](Supplementary Table S1).

### Study methods

In this study, we used in-depth interviews and key informant interviews as the primary data collection methods. These approaches are among the most widely used methods in qualitative research [19]. Due to their flexibility and ability to follow up on responses, in-depth interviews provide rich information from diverse perspectives and are suitable for in-depth exploration of cultural contexts and identification of complex social and organizational factors.

### Study setting

This study was conducted in Khash County, located in Sistan and Baluchestan Province in southeastern Iran. Khash, with a population of approximately 173,821 (based on the 2016 census), is mainly located in rural areas with high geographical dispersion. The majority of the inhabitants are Balochi and speak the Balochi language (Sarhad and Makrani dialects). The dominant religion in Khash is Sunni Hanafi, with a minority of Shiites. Khash borders Zahedan and Mirjaveh to the north, Taftan to the west, Iranshahr and Mehristan to the southwest, Sib and Soran to the south, and Saravan, Golshan, and the border with Pakistan to the east and southeast. Khash’s climate is hot and dry with an average annual rainfall of about 174.9 mm. Temperatures vary up to 40 °C in summer and − 5 °C in winter, but due to its altitude of 1415 m above sea level and proximity to Mount Taftan, it is milder than other parts of the province [20]. In this study, stray dogs refer to free-roaming dogs without identifiable owners, often found in urban and rural areas of Khash, distinct from semi-free owned dogs that move between yards and public spaces.

### Participants and recruitment

This study employed purposive sampling to recruit 41 eligible participants (29 males, 70.7%; 12 females, 29.3%) in Khash County from March 2025 to June 2025. Participants included six ordinary residents, seven family members, nine institutional stakeholders, and 19 dog bite victims, all interviewed via semi-structured interviews to elucidate social and organizational factors influencing dog bites. Including 22 participants without animal bite history (53.7%) was essential to capture diverse community perceptions, cultural norms, and institutional insights, enriching the contextual understanding of bite risks (Table 1, Supplementary Tables S3).

### Inclusion and exclusion criteria

The inclusion criteria for the study included having direct experience of dog bites or management experience related to the subject in local organizations, residing in Khash city for at least one year, willingness to participate and sign a consent form, and having an active file in health service centers for access. Exclusion criteria also included inability to communicate effectively (e.g., advanced dementia, profound hearing loss, or language barriers despite translation), unwillingness to continue the interview, or providing information unrelated to the research objectives. (Supplementary Table S2)

### Data collection tools and procedures

We collected data from participants through semi-structured interviews and face-to-face methods. The interviews were conducted in person by a local person who was familiar with the cultural and social conditions of the area (first author). A copy of the interview guide was prepared by reviewing the research literature on dog bites for ordinary people (without animal bites), animal bite victims and their families, and institutional officials (such as health and municipal officials, agricultural Jihad, veterinary medicine, and education). After a pilot study with five people, minor changes were made to the interview guide, and some questions were rewritten based on participant feedback to make them easier to answer. For example, residents were asked: “What is your experience with the presence of stray dogs?“, bitten individuals: “How did the incident occur?“, families: “How did the dog bite affect you?“, health officials: “What strategies do you implement for prevention?“, and other organizations: “What programs do you have to control stray dogs?“. The interview guides for the nine groups are provided in (Supplementary Table S3).

Forty-one semi-structured interviews (45–60 min) were conducted in local settings (health centers, mosques, homes of trusted individuals). Before the interviews, the objectives of the study were explained, and written consent was obtained. Participants were assured that the information would remain confidential and that the audio files would be deleted after implementation. Interviews were recorded. After 30 interviews, data saturation was observed; two additional interviews were conducted for certainty. Data were recorded via a mobile app, and field notes were taken for nonverbal observations. Interview guides for the five groups are provided in Supplementary Table S3.

### Trustworthiness

Trustworthiness was ensured using Lincoln and Guba’s criteria [21]. Credibility was maintained through triangulation of data from diverse participant groups (residents, bite victims, families, stakeholders) and member checking with five participants to validate findings. Transferability was supported by detailed descriptions of Khash’s context and participant demographics (Table 1, Supplementary Table S2). Dependability was achieved by maintaining an audit trail of interview transcripts, codes, and field notes, reviewed by two researchers. Confirmability was ensured through reflexivity, with researchers documenting biases and assumptions in field notes, and expert validation of the coding process by three independent researchers.

To ensure confirmability, a reflexivity statement was maintained: the research team, comprising health education and health promotion experts, acknowledged potential biases from urban-based training and mitigated them through regular critical reflection, field note documentation, and consultation with local Balochi-speaking stakeholders.

### Data analysis

The data were analyzed using a guided content analysis method based on the approach of Elo and Sigas (2008) [22] in three stages: preparation, organization, and reporting. This study utilized deductive coding within Directed Content Analysis (DCA) to align with established frameworks on social and organizational factors influencing dog bites [22]. Inductive coding was excluded because it is better suited for exploratory studies aiming to generate new theories or themes, whereas this study sought to test and refine specific, predefined categories tailored to Khash’s unique border and cultural context. Deductive coding ensured focused, context-specific insights for prevention strategies, avoiding overly broad themes [22]. In the preparation stage, the interviews were transcribed verbatim and carefully read several times to gain a deep and comprehensive understanding of their content. In the organization stage, a matrix was developed to identify key concepts, and the data was reviewed several times to extract initial codes appropriate to social and organizational factors. Meaningful units that did not fit the initial categories were either merged into existing categories or organized as new categories based on conceptual connections. In the reporting stage, the findings were described in the form of themes, subcategories, and open codes.

To ensure the accuracy of the analysis, an independent researcher reviewed the coding and categorization process, and in case of disagreement, the issue was discussed with a third researcher. It should be noted that the transcription of the interviews was done using Microsoft Word software, and data analysis was done using MAXQDA 12 software.

### Measuring demographic variables

Age and work experience were measured in years. The level of education was divided into seven categories: primary, junior high school, senior high school, diploma, bachelor’s degree, master’s degree, and doctorate. The occupation of the participants was classified into the categories of student, housewife, employee, driver, repairman, shopkeeper, and farmer. For policymakers, it was classified into the governor, responsible for non-communicable diseases, environmental health, agricultural jihad, urban services, veterinary medicine, education, health workers, and local trustees. The participants were divided into four groups based on ethnicity (Baluch and Fars), religion (Sunni and Shia), and place of residence (urban and rural). Economic status was self-reported and assessed in three categories: good, average, and poor (Table 1).

## Results

From the semi-structured interviews, 610 codes were extracted. After merging similar codes, 180 codes, 15 subcategories, and four categories were identified at both the social and organizational-political levels. At the social level, environmental and non-behavioral factors (such as the growth of the stray dog population and poor waste management), social factors (lack of public awareness about the dangers of dog bites and irresponsible dog owners, beliefs, and attitudes), and behavioral factors (high-risk behaviors, inappropriate interactions with dogs, and vulnerability of specific groups) were extracted. At the organizational-political level, managerial and organizational barriers were identified, including a lack of infrastructure, weaknesses in implementing regulations, and instability of interventions and available resources, such as local educational human resources, capacity for inter-sectoral interventions, existing administrative structure, local social demands, and implicit alignment of institutions (Table 2).

**Table 1:** Demographic characteristics of study participants

Variable	Bite Victims	Household Contacts	Unexposed Residents	Key Stakeholders
	Frequency (%)	Frequency (%)	Frequency (%)	
Mean Age (Mean, SD)	20.63 (8.54)	35 (8.12)	30 (9.25)	45 (7.14)
Marital Status				
Married	12 (63.2%)	6 (85.7%)	4 (66.7%)	9 (100%)
Single	7 (36.8%)	1 (14.3%)	2 (33.3%)	–
Gender				
Male	13 (68.4%)	4 (57.1%)	4 (66.7%)	8 (88.9%)
Female	6 (31.6%)	3 (42.9%)	2 (33.3%)	1 (11.1%)
Education Level				
Primary	5 (26.3%)	2 (28.6%)	1 (16.7%)	–
Lower Secondary	5 (26.3%)	1 (14.3%)	1 (16.7%)	–
Upper Secondary	4 (21.1%)	1 (14.3%)	1 (16.7%)	–
Diploma	4 (21.1%)	2 (28.6%)	2 (33.3%)	1 (11.1%)
Bachelor’s Degree	1 (5.3%)	–	1 (16.7%)	1 (11.1%)
Master’s Degree	–	1 (14.3%)	–	6 (66.7%)
Doctorate	–	–	–	1 (11.1%)
Ethnicity				
Baloch	15 (78.9%)	6 (85.7%)	5 (83.3%)	7 (77.8%)
Fars	4 (21.1%)	1 (14.3%)	1 (16.7%)	2 (22.2%)
Religion				
Sunni	15 (78.9%)	6 (85.7%)	5 (83.3%)	7 (77.8%)
Shia	4 (21.1%)	1 (14.3%)	1 (16.7%)	2 (22.2%)
Residence				
City	11 (57.9%)	4 (57.1%)	4 (66.7%)	8 (88.9%)
Village	8 (42.1%)	3 (42.9%)	2 (33.3%)	1 (11.1%)
Economic Status				
Good	6 (31.6%)	2 (28.6%)	2 (33.3%)	9 (100%)
Average	8 (42.1%)	3 (42.9%)	3 (50%)	–
Poor	5 (26.3%)	2 (28.6%)	1 (16.7%)	–

### Social factors

#### Environmental and non-behavioral factors

##### Growth of stray dog population

This category refers to the environmental and non-behavioral conditions leading to the increase in the population of free-ranging dogs in urban and rural areas of Khash. Easy access of dogs to garbage accumulated in the streets and alleys due to poor waste management, uncontrolled reproduction due to the lack of sterilization programs, and the movement of semi-free owner-operated dogs between yards and streets are the main factors for the growth of this population. The accumulation of garbage in the peripheral areas of cities and villages, especially in the warm seasons, provides a stable food source for dogs and leads to their increased presence in public environments.

*An informed person said*,* “In our statistics*,* rural owner-operated dogs rank first in biting*,* followed by urban owner-operated dogs; only then do stray dogs come.” [Non-communicable diseases officer*,* 41 years old*,* 17 years of experience].*

##### Waste management

Participants pointed out the problems caused by inefficient waste management in urban and rural areas, which has led to an increase in the presence of stray dogs and the risk of bites. The accumulation of waste in streets, alleys, and wastelands acts as a constant food source for stray dogs and increases their presence near residential areas. The lack of a regular waste collection system, the lack of animal-proof containers, and the neglect of cleaning public environments are the main reasons for this problem. In the peripheral areas of the city and villages, waste is sometimes not collected for several days, which attracts dogs to these areas, especially in the warm season, and increases the risk of dangerous interactions with humans, especially children.

*A dog-bitten person said*,* “If the garbage were collected on time*,* the dogs would not be roaming around the houses so much. Now the children are afraid to go out and play in the streets.” [42-year-old mother*,* rural resident*,* housewife]*.

#### Social factors

##### Lack of public awareness

Many residents, especially in underserved areas, lack sufficient information about dog body language, signs of aggression, and safe interaction methods. This ignorance leads to behaviors that provoke dogs and increase the risk of bites. The Lack of ongoing education programs in schools and health centers, combined with limited cooperation between local institutions such as councils and health workers, has exacerbated this problem.

*One informant said*,* “If school teachers and councils cooperate*,* we can better inform people. They are also concerned about dogs.” [Community Health Worker*,* 45 years old]*.

##### Irresponsibility of pet dog owners

Participants reported abandoning dogs in the streets as a factor in increasing bites. This behavior, which is common in Khash due to the lack of adequate supervision, causes dogs to roam and encounter pedestrians, especially children and the elderly, in an uncontrolled manner. Participants also cited the Lack of use of leashes as a factor in increasing the risk of bites, as dogs without leashes display unpredictable behaviors in public streets. Neglect of neutering was also reported by participants, which leads to increased aggression and roaming of dogs, resulting in bites. Management data shows that rural owner-operated dogs are the number one cause of bites.

*A key informant said*,* “Rural owner-operated dogs are our number one cause of bites.” [Environmental health officer*,* 54 years*,* 20 years of experience]*.

*An informant said: “Dog owners here do not put leashes on*,* dogs go wherever they want and…” [Mother*,* 34 years*,* housewife]*.

##### Beliefs and attitudes

Participants reported the belief in dogs as guardians as a factor in increasing bites. This belief, common in both rural and urban areas, leads residents to leave guard dogs unattended in the streets, thereby increasing the risk of uncontrolled encounters. Participants also cited the perception that guard dogs are harmless as a factor in risky behaviors, such as approaching these dogs without caution. The belief that herding dogs do not bite was also reported by participants, leading to unsafe interactions with these dogs, especially in rural areas. Trust in familiar dogs in the neighborhood, especially among children, was identified as a factor in reducing caution and increasing bites. Also, the belief that dogs protect rural homes, which leads to untrained and unchained dogs, was reported as a factor in increasing the risk of bites.

*An informant said*,* “People think that herding dogs never bite*,* but this belief has caused many bites.” [Rural District Administrator*,* 52 years old]*.

#### Behavioral factors

##### Risky interactions

Participants reported high-risk behaviors, especially among children and young people, as a factor in increasing bites. These behaviors include teasing dogs, approaching them in unsafe situations (such as when eating or defending puppies), or ignoring warning signs. Running, yelling, or making sudden movements in the presence of stray dogs can also trigger a defensive response. These behaviors are more common in densely populated urban and rural areas, where stray dogs are more common.

*A family member said*,* “Some children think dogs are their toys; they chase them or play with them. Then suddenly the dog gets angry.” [50-year-old father*,* shopkeeper]*.

##### Vulnerability of specific groups

Participants highlighted children and the elderly as groups vulnerable to the risk of dog bites. Children may tease or approach dogs out of curiosity or ignorance, which triggers a defensive reaction in the dogs. The elderly is also less able to escape or defend themselves due to reduced reaction speed or mobility problems. These groups need more supervision and targeted training.

*A family member said*,* “My father is 70 years old*,* he cannot run away quickly. The dog attacked him in the alley.” [Farmer*,* rural resident*,* 42 years old]*.

**Table 2:** Classification of social and organizational factors

Social Factors		
Category	Subcategories	Sample Open Codes
Environmental and Non-Behavioral Factors	Growth of Stray Dog Population	• Increase in stray dog numbers • Inefficiency of sterilization programs • Uncontrolled reproduction • Lack of monitoring of dog population • Migration of dogs from neighboring areas
	Waste Management	• Accumulation of waste in public areas • Lack of durable waste containers • Delay in waste collection • Dogs’ access to food resources • Scattering of waste by animals
*Social* and Behavioral Factors	Lack of Public Awareness	• Lack of knowledge about prevention methods • Insufficient education in schools • Limited public information campaigns
	Irresponsibility of Dog Owners	• Abandoning dogs in public spaces • Not using collars • Neglecting sterilization • Lack of training for dogs
	Beliefs and Attitudes	• Belief in dogs as guardians • Perception of guard dogs as harmless • Belief that herding dogs do not bite • Trust in familiar neighborhood dogs • Belief in dogs’ protection of rural homes
	Risky Interactions	• Provoking dogs with sudden movements • Approaching dogs while eating • Ignoring signs of aggression • Running in the presence of dogs • Playing with unfamiliar dogs • Ignoring dogs’ growling • Staring into dogs’ eyes • Sudden touching of dogs • Approaching unfamiliar dogs
	Vulnerability of Specific Groups	• Children’s curiosity • Limited mobility of the elderly • Children’s unawareness of dangers • Slowed reactions in elderly
Organizational Factors		
Managerial and Organizational Barriers	Lack of Infrastructure	• No dog shelter sites • Insufficient fencing • Lack of water and electricity • No security personnel
	Weak Enforcement of Regulations	• Ineffectiveness of committee decisions • Budget shortages • Lack of institutional commitment • Weak follow-up on regulations • Poor inter-departmental coordination
	Instability of Interventions	• Short-term reactive actions • Lack of sustained funding • Suspension of dog capture campaigns • Absence of long-term planning
Available Resources	Healthcare Infrastructure	• Access to health services • Availability of rabies vaccine and serum • Free medical services • Presence of trained community health workers (CHWs) • Recording cases in the Sib System
	Local Human Educational Resources	• Native teachers • Community health workers knowledgeable in Balochi language • Education delivered in local dialect • Building community trust
	Inter-sectoral Intervention Capacity	• Occasional cooperation between municipality and veterinary organization • Dog vaccination campaigns • Limited environmental cleanup
	Existing Administrative Structure	• Seasonal health committees • Written decrees from the governorate office • Intersectoral coordination
	Local Social Advocacy	• Pressure from councils for dog population management • Community requests for dog removal • Local village council meetings • Participation in dog vaccination • Demand for risk reduction for children
	Implicit Institutional Alignment	• Acceptance of the necessity for dog control • Mental readiness for cooperation • Joint institutional meetings

### Organizational factors

#### Managerial and organizational barriers

##### Lack of infrastructure

Participants cited the lack of infrastructure to manage stray dogs as a key barrier to preventing dog bites. The municipality of Khash lacks a site equipped to house, euthanize, or sterilize stray dogs, and the proposed land for this purpose lacks basic facilities such as fencing, water, electricity, and security. This lack of infrastructure has made it impossible to implement dog population control programs and has led to an increase in the presence of stray dogs in urban and rural areas. This has increased the risk of bites, especially in areas with garbage accumulation that attracts dogs, and has increased the burden on the health system to treat bite cases.

*A policymaker said*,* “We have a landfill*,* but we do not have the equipment; it’s not fenced*,* it’s just an empty lot.” [Urban service manager*,* 47 years old*,* urban resident]*.

##### Weak enforcement of regulations

Participants cited the incomplete implementation of resolutions of health and food safety committees as an obstacle to dog bite prevention. These resolutions, often approved in interdepartmental meetings involving institutions such as health, municipality, and veterinary medicine, are not fully implemented due to a lack of funding, poor follow-up, or insufficient commitment from some departments. This lack of coordination has turned preventive programs, such as collecting stray dogs or vaccination campaigns, into reactive and discontinuous measures and has reduced public trust in institutions.

*“We have a committee that all departments come to*,* report to*,* but its resolutions are not always implemented*,*” said one policymaker. [Non-communicable diseases manager*,* 41 years old*,* 17 years of experience].*

##### Instability of interventions

Participants identified the ad hoc and short-term nature of dog bite prevention programs as a key challenge. These programs, such as dog round-up campaigns or public education, are often implemented in response to complaints or an increase in bite cases, and lack ongoing funding or enforcement. This instability has perpetuated the cycle of increasing stray dog populations and bites, wasting limited resources. Interviews revealed that the lack of strategic planning and ongoing monitoring has reduced the effectiveness of interventions.

*A policymaker said*,* “Every year we launch a dog round-up program*,* but when the budget runs out*,* it is cut short.” [Veterinary Officer*,* 44 years old*,* urban resident]*.

#### Available resources

##### Healthcare infrastructure

Participants identified the availability of well-equipped health facilities as a key capacity to respond to dog bites. The presence of 13 comprehensive health centers with anti-rabies vaccines and serum has provided free or low-cost access to services for residents of Khash and even those coming from nearby areas. The presence of trained health workers has strengthened the provision of primary care and preventive education. This infrastructure plays an important role in managing bite cases by reducing economic and geographical barriers.

*A policymaker said*,* “We have thirteen centers*,* all of which have serum and vaccines. Even if someone comes from another city*,* we provide services here.” [Non-communicable diseases officer*,* 41 years old*,* 17 years of experience].*

##### Local human educational resources

Participants emphasized the role of teachers, health workers, and local village heads in dog bite prevention education. With their linguistic and cultural knowledge, these individuals have increased the acceptance of training in rural and nomadic areas. The use of the Balochi dialect and local examples made the prevention messages more understandable.

*A policymaker said*,* “Our health workers are Baloch and they are comfortable talking to people. In the villages*,* people listen better when it is in their language.” [Non-communicable diseases officer*,* 41 years old*,* 17 years of experience].*

##### Intersectoral intervention capacity

Participants noted the history of collaboration between institutions such as the municipality, veterinary medicine, and Jihad Keshavarzi, which has the potential for broader coordination. These ad hoc collaborations, such as dog vaccination, have had positive results but have remained limited due to instability.

*A policymaker said*,* “In one of the sectors*,* Jihad Keshavarzi and Veterinary Medicine came to vaccinate dogs*,* and we also provided statistics from the health center.” [Non-communicable diseases officer*,* 41 years old*,* 17 years of experience].*

##### Existing administrative structure

Participants highlighted the health and food safety committees in the governorate as a platform for shared decision-making. These structures enable the implementation of prevention programs through coordination and written approvals; however, a lack of funding has limited their implementation.

*A policymaker said: “The health committee meets every quarter*,* all institutions come and we plan for stray dog control*,* but the budget is limited.” [Governorate expert*,* 50 years old]*.

##### Local social advocacy

Participants noted the coordinated efforts of councils, village councils, and residents to demand preventive measures against stray dogs. This demand has created positive pressure on responsible institutions such as the municipality and the health network, especially in areas where children are at risk of being bitten by stray dogs. By holding local meetings and conveying people’s concerns, the councils and village councils have played a key role in drawing the attention of institutions to the issue. This advocacy has increased community participation in prevention programs, such as requiring the vaccination of owned dogs or collecting stray dogs. It has helped reduce high-risk behaviors, such as abandoning dogs in the streets.

*One informed person said*,* “People themselves came to ask why the municipality doesn’t catch dogs? The councils have also been very proactive.” [Rural District Administrator*,* 52 years old]*.

##### Implicit institutional alignment

Participants noted the mental readiness of key institutions such as the health network, the municipality, the veterinary service, and the Agricultural Jihad to cooperate in controlling stray dogs. This alignment, despite facing obstacles like insufficient funding and coordination issues, has the potential to develop joint and sustainable programs. Interviews revealed that institutions have accepted the need to regulate stray dogs as a public health priority and have shown a willingness to share information and plan together in health committee meetings. This readiness can be translated into practical and effective measures by strengthening legal frameworks and allocating resources.

*A policymaker said*,* “If the governor issues a decree*,* all the agencies will cooperate. But without it*,* nothing will work.” [Urban services manager*,* 43 years old]*.

## Discussion

Dog bites represent a critical public health issue in Iran, particularly in Khash, an endemic region for rabies, due to their significant physical, psychological, and social consequences, including the risk of rabies transmission. This study systematically examines the social and organizational factors driving dog bites, offering insights into the complex interplay of environmental, cultural, behavioral, and institutional elements that exacerbate this issue in Khash. By analyzing these factors, the study provides a foundation for developing targeted interventions to mitigate dog bite incidents and their associated risks.

At the social level, dog bites in Khash are influenced by a combination of environmental, cultural, and behavioral factors. Environmental and non-behavioral factors include the uncontrolled growth of stray dog populations and poor waste management practices. The accumulation of garbage in rural and marginal areas, particularly during warm seasons, provides a consistent food source for stray dogs, attracting them to residential zones and increasing the likelihood of human-dog interactions [23, 24]. The absence of durable waste containers and irregular collection systems exacerbates this issue, drawing dogs into populated areas where children, who frequently play in open spaces, are particularly vulnerable [25]. Cultural factors, such as low public awareness about dog bite risks and the irresponsibility of dog owners, further aggravate the problem. Traditional norms in Khash, particularly in rural areas, promote keeping semi-free dogs as guardians of livestock or property, which fosters risky interactions between humans and dogs [26]. These dogs, often left unsupervised, contribute to bite incidents due to their proximity to human populations.

Behavioral factors also play a significant role, particularly among vulnerable groups like children. The low mean age of bite victims underscores children’s susceptibility, driven by their natural curiosity, frequent outdoor play, and lack of safety education regarding dog interactions [27]. In rural Khash, where dog management is inadequate, children are at heightened risk due to their inability to recognize or respond to dogs’ defensive behaviors, such as growling or territorial posturing. Studies by Meints et al. [28] and Walsh et al. [29] confirm that children often misinterpret dog body language, engaging in risky behaviors like teasing, staring into dogs’ eyes, or approaching them abruptly, which can provoke defensive or aggressive responses. Adolescents also exhibit high-risk behaviors, such as yelling, running, or approaching dogs during feeding or while they protect their puppies, further elevating bite risks.

The predominance of male victims (57.1%–88.9%) reflects traditional gender roles, where men are more likely to engage in outdoor occupations like agriculture or herding, increasing their exposure to stray or semi-free dogs [30]. The lack of educational programs in rural schools perpetuates ignorance about safe dog interaction, particularly among students, amplifying the risk of bites [31]. Cultural beliefs, deeply rooted in Khash’s rural communities, exacerbate these risks. The perception of dogs as harmless guardians of property or livestock reduces residents’ vigilance, particularly among children, who may approach even familiar dogs without caution [32]. Oxley et al. [33] note that many bites occur from familiar dogs, often perceived as “accidental” or “unintentional” by victims, especially when the dog belongs to the household or neighborhood, further undermining preventive behaviors.

At the organizational level, ineffective waste management, lack of sterilization programs, and the movement of semi-free dogs significantly contribute to the stray dog population in Khash. Poor waste management, characterized by garbage accumulation in streets and wastelands, particularly in underserved areas, creates an environment conducive to stray dog proliferation [34, 35]. The absence of robust waste containers and regular collection systems, combined with the geographical dispersion of rural areas, complicates waste and dog population management. The lack of sterilization programs allows uncontrolled dog reproduction, while semi-free dogs, often kept for protection under local cultural norms, pose a greater risk than fully stray dogs due to their frequent interactions with humans. Bay et al. [5] corroborate this pattern, highlighting how environmental factors directly amplify bite incidents in rural Iran.

The lack of basic infrastructure further hinders effective dog population control. Khash lacks dedicated dog shelters, fencing in high-risk areas, and essential utilities like water and electricity for potential boarding sites. The absence of trained personnel to monitor stray dogs exacerbates these challenges. Petrean et al. [36] in Romania similarly report that inadequate infrastructure and lack of trained staff create unfavorable conditions for dogs and increase human risks. Additionally, Rault et al. [37] highlight how insufficient material resources, such as monitoring equipment and budgets, limit the enforcement of animal control laws, a challenge mirrored in Khash.

Weak implementation of local committee resolutions presents another significant barrier. In Khash, ineffective resolutions, insufficient funding, lack of institutional commitment, poor follow-up, and inter-agency incoordination—particularly between municipalities, veterinary services, and health networks—hinder preventive measures. Rock et al. [38] in Calgary, Canada, demonstrate that such incoordination undermines the “One Health” approach, which requires integrated efforts across sectors. Similarly, Taylor et al. [39] identify funding shortages as a significant obstacle in low-income countries, where high sterilization costs and lack of sustainable budgets limit program viability. In Khash, short-term, reactive interventions, such as temporary dog collection campaigns, lack continuity due to insufficient funding and planning, reducing their impact. Daigle et al. [40] note that short-term veterinary clinics for vaccination and sterilization often fail to achieve lasting results due to inconsistent planning and low community acceptance.

Khash’s healthcare infrastructure, comprising 13 health centers, access to free rabies vaccines and serum, trained health workers, and case registration in the SIB system, facilitates rapid bite management. However, low public awareness limits the optimal utilization of these resources. Residents, particularly children, often lack knowledge about critical preventive measures, such as immediate wound washing or seeking prompt medical attention [41]. Swedberg et al. [42] in the Philippines highlight the importance of accessible bite treatment centers and free vaccine distribution, aligning with Iran’s infrastructure. In contrast, Kisaka et al. [43] and Changalucha et al. [44] in Uganda and Tanzania, respectively, report that high costs and supply chain issues restrict access to rabies immunoglobulin and vaccines, a challenge less prevalent in Iran due to free services. However, Tulloch et al. [45] in the UK note higher adult hospitalizations and significant treatment costs (£70 million annually), contrasting with Iran’s focus on children as a high-risk group and free service provision.

The indigenous workforce in Khash, including Balochi-speaking teachers and health workers, offers significant potential for raising awareness and building community trust through education in local dialects. However, the lack of continuous training programs limits this capacity. Ma et al. [4] demonstrate that community-based dog population management, leveraging local workers, increases sterilization rates and reduces bites. Conversely, Riley et al. [46] find that while community-based training enhances program acceptance, it does not necessarily reduce bite incidents, underscoring the need for comprehensive strategies.

Inter-sectoral interventions in Khash, such as limited municipal-veterinary cooperation for dog collection and vaccination campaigns, show promise but are hampered by insufficient funding and coordination. Lushasi et al. [47] in Tanzania illustrate the efficacy of integrated bite management through health and veterinary collaboration, using rapid diagnostic tests and risk notifications, though resource constraints limit scalability. In Khash, sporadic waste cleanup efforts, aimed at reducing attractants for stray dogs, have minimal impact due to infrastructural and coordination deficits.

Khash’s administrative framework for dog bite control comprises seasonal health committees overseeing vaccination and sterilization, governorate approvals providing legal and financial support, and inter-sectoral coordination among health, veterinary, municipal, and NGO sectors. This structure has the potential for effective management if consistently activated [42]. Local social demand, driven by community councils and public concerns about child safety, pressures authorities for swift action. Village council meetings serve as platforms for voicing these concerns, and voluntary community participation in vaccination programs reduces costs and enhances effectiveness. However, tensions between public demands for quick fixes (e.g., dog culling) and ethical, scientific approaches necessitate education and mediation by local institutions. Duncan-Sutherland et al. [48] emphasize that community engagement, including advocacy for laws like collaring and fencing, is critical to successful bite prevention. Institutional alignment in Khash, evidenced by joint meetings and shared recognition of the issue, provides a foundation for coordinated policies, but Sebastian et al. [49] caution that alignment alone is insufficient without actions tailored to institutional dynamics.

## Strengths and limitations

This study offers a new perspective by emphasizing Baloch cultural norms and semi-free dogs, and analyzing organizational-political factors in the border region. The context of Khash, with its ethnic, geographical, and border characteristics (close to Pakistan), and its focus on the complex interactions of social and organizational factors, distinguishes this study from a methodological and conceptual perspective. From a theoretical perspective, this study, using a biosocial approach, emphasizes the necessity of multilevel analysis of public health issues, demonstrating the dynamic interaction of cultural, behavioral, and institutional factors in shaping dog bites.

From a practical perspective, the findings suggest localized solutions such as Baloch dialect-based training, strengthening inter-institutional coordination, and reviewing local policies with sustainable budget allocation that apply to border regions with similar challenges. Limitations of the study include focusing on a specific region and relying solely on qualitative methods, which limit generalizability. However, the depth of analysis of the local context and conceptual framework compensates for this limitation. Future research could strengthen the generalizability and effectiveness of interventions by using mixed (qualitative-quantitative) approaches, statistical modeling of the identified factors, and examining similar regions. Ultimately, this study contributes to reducing the burden of dog bites and improving public health in deprived and border areas by providing deep insights and a unified framework, paving the way for evidence-based policymaking.

## Conclusions

This study elucidates key social factors (low public awareness, irresponsible dog ownership, and Balochi cultural beliefs promoting semi-free dogs for livestock protection) and organizational barriers (inter-institutional incoherence, inadequate infrastructure, and unstable interventions) driving dog bites in Khash. Facilitators include robust healthcare infrastructure, local advocacy, and inter-sectoral collaboration potential. These findings address the research questions, highlighting social factors (beliefs, attitudes, risky behaviors) and organizational challenges (incoherence, infrastructure deficits) that limit policies like vaccination programs. They inform evidence-based, culturally tailored interventions—such as Balochi-language education, enhanced waste management, and sustained inter-agency coordination—to reduce bite incidents and rabies risks. This framework benefits public health officials, policymakers, veterinary services, and community leaders in border regions, fostering sustainable dog bite prevention strategies and improving community safety.

## Supplementary Information

Supplementary Material 1.

Supplementary Material 2.

Supplementary Material 3.

## Data Availability

The qualitative data supporting the findings of this study are included within the article in the form of categorized themes and sub-themes.
