# Supplementary material for: Exploring the social and organizational factors influencing dog bites: a qualitative study
Source: BMC Public Health. 2026 Jan 2;26:432. doi: 10.1186/s12889-025-26083-9 (PMC12865953; doi:10.1186/s12889-025-26083-9)
Supplement: Supplementary file 2 — Supplementary Material 2. [file 12889_2025_26083_MOESM2_ESM.docx]

| **Group** | **Inclusion Criteria** | **Exclusion Criteria** |
| --- | --- | --- |
| **General population (no history of animal bite)** | - Permanent resident of Khash County (minimum continuous stay of 6 months) - Aged 18 years or older - Ability to communicate in Balochi or Persian - Awareness of the presence of stray dogs in the neighbourhood - Willingness to participate and provision of written informed consent | - History of any animal bite in the past 5 years - Unwillingness to participate - Inability to communicate (e.g., severe cognitive or language impairment) - Temporary residence or less than 6 months of stay in Khash |
| **Dog bite victims** | - Documented history of dog bite (stray or owned) within the past 2 years - Bite incident recorded in the national “SIB” health information system or in Khash health facilities - Ability to provide detailed account of the incident - Willingness to participate and provision of written informed consent | - Bite caused by animals other than dogs - Lack of access to validated medical records - Inability to communicate - Unwillingness to participate or recall the incident |
| **Family members of dog bite victims** | - Parent, spouse, or legal guardian of a dog bite victim - Awareness of the bite incident and its personal or household impacts - Permanent resident of Khash County (minimum 6 months) - Willingness to participate and provision of written informed consent | - No direct relationship with the bite victim - Unwillingness to participate - Inability to communicate - Temporary residence or less than 6 months in Khash |
| **Institutional stakeholders** | - Employment in institutions relevant to dog bite prevention or rabies control - Minimum 1 year of professional experience in Khash County - Awareness of institutional programmes for dog bite or rabies prevention - Ability to provide accurate organisational information - Willingness to participate and provision of written informed consent | - No direct involvement in dog bite prevention programmes - Less than 1 year of experience in Khash - Unwillingness to participate - Inability to provide accurate organisational information |

Table S2. Inclusion and Exclusion Criteria for Participant Groups
