# Supplementary material for: Exploring the social and organizational factors influencing dog bites: a qualitative study
Source: BMC Public Health. 2026 Jan 2;26:432. doi: 10.1186/s12889-025-26083-9 (PMC12865953; doi:10.1186/s12889-025-26083-9)
Supplement: Supplementary file 3 — Supplementary Material 3. [file 12889_2025_26083_MOESM3_ESM.docx]

Table S3. Interview Guide for Qualitative Study on Dog Bites

| **Group** | **Starter Question** | **Semi-Structured Questions** | **Probing Questions** | **Closing Question** |
| --- | --- | --- | --- | --- |
| General Public (without animal bite history) | What is your experience with the presence of stray dogs in your neighborhood? | 1. Have you witnessed stray dog attacks on people in your neighborhood? 2. Which areas in your neighborhood are most exposed to stray dogs? 3. Have you changed your routes or activity times due to the presence of stray dogs? 4. What measures do you take to protect yourself and your family from stray dogs? 5. Do you know what to do when encountering aggressive dogs? 6. Have you received any training on dog bite prevention? From what source? 7. What actions do you think local authorities should take to control stray dogs? 8. What is your opinion on local policies and programs for dog bite prevention? 9. How do the characteristics of your living environment affect the increase or decrease in dog bites? | • Why do you take these measures? • What barriers exist to implementing preventive actions? • What factors make you feel at risk? | Based on your experience, what suggestions do you have to reduce dog bite incidents in the region? |
| Dog Bite Victims | Please describe your experience with the dog bite incident. | 1. When, where, and under what circumstances did the dog bite incident occur? 2. What information do you have about dealing with aggressive dogs, and how did you acquire it? 3. What actions did you take immediately after the dog bite? 4. Where did you seek treatment, and how was the treatment and follow-up process? 5. How has this incident affected your daily life (physically, psychologically, socially)? 6. Has your behavior or attitude toward dogs changed after this incident? 7. What is your opinion on health centers’ programs for dog bite prevention? 8. What is your opinion on local policies and programs for dog bite prevention? 9. How do the characteristics of your living environment affect the increase or decrease in dog bites? | • Why did you take these actions? • What barriers did you face during treatment or prevention? • How did you feel at that moment, and why? | Based on your experience, how can dog bite incidents in the region be reduced? |
| Family Members of Dog Bite Victims | How has the dog bite incident affected your family? | 1. Has the dog bite incident caused changes in your family’s daily routine? 2. How have the costs of treatment and follow-up for the dog bite affected your family’s finances? 3. Have you received any support from local authorities? What kind of support? 4. What measures have you taken to protect other family members from dog bites? 5. Is sufficient information on dog bite prevention and management provided to families? 6. What are your expectations from local authorities to prevent such incidents? 7. What is your opinion on health centers’ programs for dog bite prevention? 8. How do the characteristics of your living environment affect the increase or decrease in dog bites? | • Why did these changes occur in family life? • What barriers exist to receiving support or information? • What topics in prevention education need more attention? | Based on your experience, how can dog bite incidents in the region be reduced? |
| Health Officials | What actions do you take to prevent dog bites at the community level? | 1. What strategies are implemented to prevent dog bites in the community? 2. What challenges do you face in implementing dog bite prevention programs? 3. What educational programs are conducted to raise public awareness about dog bite prevention? 4. How is your collaboration with other organizations regarding dog bite prevention? | • Why do these challenges exist? • What barriers do you observe in collaborating with other organizations? • What factors facilitate or limit program implementation? | Based on your experience, how can dog bite incidents in the region be reduced? |
| Agricultural Jihad | What actions have you taken to manage conflicts between stray dogs and livestock? | 1. What programs exist to educate livestock owners about preventing stray dog attacks? 2. What challenges do you face in implementing dog bite prevention programs? | • Why do these challenges exist? • What barriers do you observe in educating livestock owners? | Based on your experience, how can dog bite incidents in the region be reduced? |
| Veterinary Organization | What programs do you have to control the stray dog population to reduce dog bites? | 1. What actions do you take for sterilization and vaccination of stray dogs? 2. How is your collaboration with the municipality in managing stray dogs? 3. What challenges do you face in implementing dog bite prevention programs? | • Why do these challenges exist? • What barriers do you observe in collaborating with other organizations? | Based on your experience, how can dog bite incidents in the region be reduced? |
| Municipality and Village Administration | What strategies do you have for managing stray dogs and reducing dog bites? | 1. What programs are implemented for sterilization and vaccination of stray dogs? 2. How is your collaboration with other organizations in managing stray dogs? 3. What challenges do you face in implementing dog bite prevention programs? | • Why do these challenges exist? • What factors facilitate or limit program implementation? | Based on your experience, how can dog bite incidents in the region be reduced? |
| Education Department | What actions do you take to educate students about dog bite prevention? | 1. Are there materials in textbooks about safely interacting with dogs? 2. What measures are in place to ensure student safety from stray dogs in and around school premises? | • Why have these actions been taken or not taken? • What barriers exist to implementing educational programs? | Based on your experience, how can dog bite incidents among students and in the region be reduced? |
